# Retinol and α-Tocopherol in the Breast Milk of Women after a High-Risk Pregnancy

**DOI:** 10.3390/nu9010014

**Published:** 2017-01-01

**Authors:** Reyna Sámano, Hugo Martínez-Rojano, Rosa M. Hernández, Cristina Ramírez, María E. Flores Quijano, José M. Espíndola-Polis, Daniela Veruete

**Affiliations:** 1 Departamento de Nutrición y Bioprogramación, Instituto Nacional de Perinatología, Secretaría de Salud Montes Urales 800, Miguel Hidalgo, Lomas Virreyes, Ciudad de México CP. 11000, Mexico; ssmr0119@yahoo.com.mx (R.S.); rmh080868@yahoo.com (R.M.H.); crisra07@yahoo.com.mx (C.R.); maru_fq@yahoo.com (M.E.F.Q.); 2 Departamento de Posgrado e Investigación, Escuela Superior de Medicina del Instituto Politécnico Nacional, Plan de San Luis y Díaz Mirón s/n, Colonia Casco de Santo Tomas, Delegación Miguel Hidalgo, Ciudad de México CP. 11340, Mexico; 3 Coordinación de Medicina Laboral, Instituto de Diagnóstico y Referencia Epidemiológicos (InDRE) “Dr. Manuel Martínez Báez”, Secretaría de Salud Francisco de P. Miranda 177, Lomas de Plateros, Ciudad de México CP. 01480, Mexico; 4 Departamento de Nutrición Humana, Universidad del Altiplano, Mirasol 1, Tlacomulco, Tlaxcala de Xicohténcatl CP. 90102, Mexico; jjmartinezr@yahoo.com; 5 Universidad del Valle de México, campus Chapultepec Av. Constituyentes No. 151, Miguel Hidalgo, San Miguel Chapultepec I Secc, Ciudad de México CP. 11850, Mexico; analilia_000@hotmail.com

**Keywords:** breast milk, liposoluble vitamins, high-performance liquid chromatography, pregestational obesity

## Abstract

Background: There is scant information about whether, after a high-risk pregnancy, breast milk provides enough vitamins for assuring satisfactory bodily reserves in newborns. Objective: To comparatively evaluate, in women with high-risk and normal pregnancy, the concentration of retinol and α-tocopherol in breast milk. Methods: This cross-sectional, analytical study was evaluated with reverse-phase high-performance liquid chromatography (HPLC). Informed consent was signed by 95 mothers with a high-risk pregnancy and 32 mothers with a normal pregnancy. From the mothers with a high-risk pregnancy were obtained: 23 samples of colostrum, 24 of transitional milk, and 48 of mature milk. From the normal pregnancy group, 32 mature milk samples were collected. Pregestational Body Mass Index (BMI) and the gestational weight gain were noted. Models of logistic regression were constructed to identify the variables related to a low concentration of either retinol or α-tocopherol in breast milk. Results: The concentration of retinol and α-tocopherol in mature milk was 60 (interquartile range (IQR), 41–90) and 276 (103–450) μg/dL, respectively, for the high-risk pregnancy group, and 76 (65–91) and 673 (454–866) µg/dL, respectively, for the normal pregnancy group (*p* = 0.001). The concentration of retinol and α-tocopherol was similar in the subgroups of mothers with different disorders during gestation. A clear correlation was found between a greater pregestational weight and a lower concentration of retinol (*Rho* = –0.280, *p* = 0.006), and between α-tocopherol and retinol in all cases (*Rho* = 0.463, *p* = 0.001). Among women having a high-risk pregnancy, those delivering prematurely rather than carrying their pregnancy to term had a reduced concentration of retinol (54 (37–78) vs. 70 (49–106) µg/dL; *p* = 0.002) and a tendency to a lower concentration of α-tocopherol in breast milk (185 (75–410) vs. 339 (160–500) µg/dL; *p* = 0.053). Compared to mothers with a normal pregnancy, those with a high-risk pregnancy (whether carried to term or ending in preterm delivery) exhibited a reduced concentration of retinol in mature milk (*p* = 0.003), as well as a tendency to a lower concentration of α-tocopherol (*p* = 0.054). Conclusion: Even though the women in the high-risk pregnancy group showed a deficiency of vitamins A and E in their breast milk, the unique biological benefits of this milk justify the promotion of breast feeding as the optimal method of nourishing neonates and infants. In these cases, it should be recommended that the woman increase her consumption of certain nutrients during pregnancy. Additionally, after childbirth mothers should consider the use of supplements to produce milk of adequate quality and thus meet the needs of the baby and prevent any deficiency in micronutrients.

## 1. Introduction

Approximately 22% of all pregnancies are high-risk in Mexico each year [1]. One of the risk factors for complications during pregnancy is being overweight, leading to a higher prevalence of gestational diabetes, hypertension, pre-eclampsia, asthma, and thromboembolic disease [2]. Around 60% of Mexican women in reproductive age are overweight [3]. Additionally, excessive weight gain during pregnancy also increases the risk of adverse outcomes for both mother and child [4], and a high percentage of Mexican women gain more weight than recommended [5].

Similarly, gestational diabetes is a risk factor for the increased incidence today of teratogenicity, which has been observed more frequently in fetuses of diabetic versus healthy mothers. These malformations are the result of tissue injury caused by the activity of free radicals [6]. Natural antioxidants reduce the adverse effects of these radicals, since they can capture and neutralize reactive oxygen species, thus preventing lipid peroxidation. This neutralization is essential, especially in situations where oxidative stress is elevated, such as gestational diabetes and preeclampsia [7]. One of the essential natural antioxidants is vitamin E, which comprises a group of eight fat-soluble compounds that are classified as α-, β-, γ-, and δ-tocopherol or tocotrienol. Of these, α-tocopherol is the most active compound [8].

The variation of glycaemia during pregnancy leads to serious complications for the mother-child binomial [7,8]. Although there is currently little evidence of the association between the concentration of retinol and diabetes in pregnant women [9], this disease makes such women more prone to a marginal or poor biochemical status of vitamin A [9]. In addition to potentiating the complications caused by diabetes in pregnant women, low levels of vitamin A leave their children vulnerable to developing a deficiency of the same [10,11].

An adequate nutritional status during the first 2–3 years of life is necessary for subsequent health and well-being. Deficient development cannot be reversed after passing this critical window [12]. In this sense, a study in Egypt stressed that vitamin E is a key micronutrient for development, with its deficiency leading to a delay in growth [13]. The authors also noted that 78.2% of children showing a delay in their growth were deficient in vitamin E, having plasma concentrations of α-tocopherol at 7.7 mmol/L versus 14.1 mmol/L for the group of control children [13]. Fares et al. [14] revealed that deficiencies of vitamins A, E, and D were very common among infants with very low birth weight in Tunisia, and were associated with preeclampsia. However, the risk of preeclampsia did not diminish after the administration of vitamin C and E in a series of studies in Western countries [15,16].

The results of administering vitamin E supplements have been variable. One reason is that the appropriate status of α-tocopherol during pregnancy has not yet been defined. Moreover, in the various global reports on the usefulness of vitamin E supplements during pregnancy, the plasma concentration of α-tocopherol is often not even determined. In situations where a low concentration of α-tocopherol was documented, the administration of supplements of vitamin E has proven beneficial. In a Hungarian population, for instance, supplementation of vitamin E in women was associated with a decreased incidence of premature babies [17]. The establishment of a clear criteria for adequate levels of vitamin E deserves further investigation.

It is also known that placental transfer of vitamins A and E during pregnancy is limited and that the reserve of these micronutrients in the newborn is low, especially in the case of premature babies. Consequently, exclusive breastfeeding is the only way to meet their nutritional needs [8]. Since exposure to hyperoxia at birth increases the risk of free radical formation [18], it is essential that the mother provide the infant a sufficient supply of vitamin E through breastfeeding.

Due to the importance of vitamins A and E for the mother and child, the aim of the present study was to determine the concentration of retinol and α-tocopherol in the breast milk of mothers after undergoing a high-risk versus normal pregnancy, as well as after giving birth preterm versus carrying their pregnancy to term. Associations were examined between low levels of vitamins A and E in breast milk and maternal characteristics.

## 2. Material and Methods

### 2.1. Study Design and Population

A cross-sectional and analytical study was conducted to determine the presence of retinol and α-tocopherol in the breast milk of 95 mothers with a high-risk pregnancy (and whose newborns were confined in the neonatal intensive care unit during the first and second month after birth) and 32 mothers with a normal pregnancy and healthy newborns. The study took place from January 2013 to October 2016. It was conducted at the Instituto Nacional de Perinatología (National Institute of Perinatology) and the School of Medicine, National Polytechnic Institute, in Mexico City.

The sample size was calculated with a 95% confidence interval, utilizing information from previous reports that estimated the mean content of vitamin A in breast milk [19]. After the mothers had agreed to participate and signed informed consent, they were asked to fill out a survey to provide data about their obstetrical background.

The manner of sampling mothers was not probabilistic or based on consecutive cases. Inclusion criteria were that the women lived in the metropolitan area of Mexico City, agreed to provide all data requested through a survey (maternal age, sociodemographic characteristics, medical and obstetrical history, course of current pregnancy and term, mode of delivery, pregnancy-related complications, weight, and complications of the neonates), were breastfeeding their newborns/infants and secreting breast milk in adequate quantities, were 18–45 years old, and were not taking medications or vitamin/multivitamin supplements. All participating mothers had been eating customary Mexican food during pregnancy and none had adopted special diets.

The 127 mothers that participated in the study were recruited by the nutrition team of the National Perinatology Institute (all women in this unit who consented were included). In the high-risk pregnancy group, all the newborns and some of the women were hospitalized. Contrarily, in the normal pregnancy group, none of the infants were hospitalized. Among the 95 mothers with a high-risk pregnancy, 48 delivered preterm and 47 carried their pregnancy to term. All 32 mothers with a history of normal pregnancy carried their pregnancy to term.

Preterm childbirth was defined as a gestation lasting less than 37 weeks. A normal pregnancy was defined as the lack of complications or disorders before and/or during gestation. When there were pregnancy-related complications, illnesses, or events that represented a health risk, the situation was considered high risk. Especially important was any appearance of the woman or an increase in complications that could have caused maternal/perinatal morbidity/mortality. The following conditions were examined for any possible association with low levels of retinol or α-tocopherol: (i) multiple gestation (involving twins, triplets, etc.), which increases the risk of infants being born prematurely (before 37 weeks of pregnancy); (ii) pregnancy with triplets or quadruplets, which implies a greater the probability of delivery by cesarean section; (iii) pregnancy after age 30 and/or after taking fertility drugs (both related to multiple births); and (iv) gestational diabetes, preeclampsia or eclampsia.

Preeclampsia, a syndrome marked by a sudden rise in the blood pressure of a pregnant woman after the 20th week of pregnancy, can affect the kidneys, liver, and brain. When left untreated, the condition may result in long term health problems or be fatal for the mother and/or the fetus. Eclampsia is a more severe form of preeclampsia, marked by anemia, seizures, and coma in the mother (implying an increased risk for a first-time pregnancy in women over 35 years of age). Preeclampsia and gestational diabetes were based on the criteria of the American College of Obstetricians and Gynecologists [20,21].

### 2.2. Milk Sampling

Samples of breast milk were aseptically obtained from the mothers, either at home or in the hospital, during the morning (08:00–10:00) and after 8–10 h of fasting. The first sample of breast milk from mothers with a history of high-risk pregnancy was obtained in the hospital during the 48 h immediately after birth. These samples were considered as colostrum. Transitional milk was obtained from these same mothers at eight days postpartum. Mature milk samples were obtained from both groups of women between the first and second month postpartum. Breast milk was delivered by mothers to the hospital milk bank with the high-risk pregnancy group, because the newborns were in the Neonatal Intensive Care. This milk was deposited in sterile polypropylene bottles and stored at 4 °C or colder. The women with a history of normal pregnancy delivered their sample of breast milk to the nutritionist when they brought their healthy child to the hospital. From each recipient, an aliquot of 15 mL was filled without leaving any air pockets (to avoid oxygenation). This aliquot was taken from a full breast sample, previously homogenized. Each aliquot was protected from ultraviolet light with aluminum foil and placed in refrigeration at –20 °C.

### 2.3. Type of Milk

From the high-risk pregnancy group, 23 of the breast milk samples obtained were considered as colostrum, 24 transitional milk, and 48 mature milk. From the normal pregnancy group, 32 mature milk samples were obtained. Colostrum is considered as the breast milk from delivery to approximately five days postpartum, transitional milk from day 6–15 postpartum, and mature milk from day 15 onwards.

### 2.4. Laboratory Methods

The procedure for determining the concentration of vitamins A and E (as retinol and α-tocopherol) was based on high-performance liquid chromatography (HPLC) in the reverse phase. The internal standard was all-trans-retinyl-acetate (800-Across-01. Software Total Chrome, version 6.3.2.0646, Whippany, NJ, USA). This determination was carried out on all of the breast milk samples from the 127 participating mothers.

Retinol and α-tocopherol levels were determined in breast milk by using a binary HPLC pump (Flexar model, Perkin-Elmer, Naperville, IL, USA) with an injector (loop size 100 μL, Flexar model), a sensitive UV7 detector (Flexar model, Norwalk, CT, USA), a three-column microbondapak rp-18 Pecosphere C-18 of 3 μm and 33.46 mm (Perkin-Elmer), and an acrodisc CR-13 mm syringe filter with a 0.2 μm PTFE (polytetrafluoroethylene) membrane (Teflon).

After the extraction, vitamins A and E were isolated from the samples of breast milk. First the matrix was saponified, followed by extraction of the analytes of interest. The esters of retinal were hydrolyzed to retinol by saponification. To 15 mL of breast milk was added 24 mL of potassium hydroxide in a solution of 16% methanol (weight per volume). This alkaline mixture was heated to 75 °C and maintained at that temperature for 30 min, then cooled for 5 min. For the extraction of the vitamins, 16 mL of petroleum ether was added in a separatory funnel, and then the solution was stirred and left to stand for 30 min. The aqueous phase was drained and disposed of three times. The etheric dissolution was washed with deionized water to eliminate the alkaline excess, reaching pH 7 (measured with pH paper). The solvent was recovered in Falcon tubes of 50 mL, to be evaporated in a steam bath at 70 °C. The residue was dissolved with 2 mL of ethanol, then filtered with acrodisk CR 13 mm syringe filters with a 0.2 μm PTFE membrane. An injection volume was taken from this solution for the chromatography with 50 µL, which was run for 10 min. To obtain the target of the reactives, a process of saponification and extraction was carried out in the absence of breast milk. All samples were processed in duplicate and each replicate was injected twice [22].

Microbondapak columns with a UV/visible detector were utilized to establish the conditions for the chromatographic system, with methanol/water (96:4) for the mobile phase and a wavelength of 325 nm for retinol and 290 nm for α-tocopherol. The analytic parameters for linearity were *R*^2^ = 0.9955 for retinol and *R*^2^ = 0.9808 for α-tocopherol, while the detection and quantification limits were 1.1 µg/dL and 2.7 µg/dL for retinol and 0.9 µg/dL and 2.3 µg/dL for α-tocopherol. Regarding accuracy, relative standard deviation (RSD) was 4.5% for the same day and RSD 4.8% between days for retinol, and 4.9% for the same day and 4.1% between days for α-tocopherol. The recovery was 85.8 ± 7.8% µg/dL for retinol and 98 ± 1.9% µg/dL for α-tocopherol. Once validated, the method was applied to the quantification of retinol and α-tocopherol in samples of breast milk [23].

### 2.5. Evaluating Maternal Body Mass Index (BMI) and Morbidity in Mothers and Newborns/Infants

The pregestational weight and height of the mother was obtained from the clinical history. The BMI was calculated considering ≤18.49 kg/m^2^ as underweight, 18.50–24.99 kg/m^2^ as normal, 25–29.9 kg/m^2^ as overweight and ≥30 kg/m^2^ as obese. The evaluation of the gestational weight was based on both the pregestational BMI and the tables of the Institute of Medicine (IOM, Washington, DC, USA). The increase in gestational weight was calculated by subtracting the pregestational weight from the maximum gestational weight, and the result was classified as adequate or below/above the recommended level, according to the guidelines of the IOM [24]. The absence or presence of morbidity in mothers and newborns/infants was determined by reviewing the corresponding clinical history.

## 3. Statistical Analysis

For clinical data, anthropometric and sociodemographic characteristics of the mothers and newborns were reported as the mean ± Standard Deviation (SD) and the median with an interquartile range (IQR) of p25–p75. Due to the nature of the variables, a parametric analysis was performed with the Student’s *t*-test and a non-parametric analysis with the Mann-Whitney *U* test and the Kruskal-Wallis test. Additionally, frequency and percentages for categorical variables were analyzed with the Pearson’s *X*^2^ test. For qualitative variables, such as marital status, occupation, level of education, and pregnancy outcome, frequency measurements were calculated. For numerical variables, including the age of the mother and pregestational weight and height, measurements of central tendency and dispersion were utilized. With the Kruskal-Wallis test, analysis was made of the difference between the concentration of retinol and α-tocopherol between groups and in relation to the pregestational BMI of the mother. For the concentration of the vitamins and the outcome of the pregnancy, the Mann-Whitney *U* test was employed. We used the median value for women with high-risk or normal pregnancy as a comparison group. The two values used in the logistic regression model were considered risk variables when they were below these medians. A statistical significance was considered at *p* < 0.05. Data entry and analysis were performed with the statistical program SPSS version 20 for Windows (SPSS Inc., Chicago, IL, USA).

## 4. Ethical Aspects 

Data gathering and analysis was confidential, taking such ethical questions as autonomy and security into account. The guidelines of the Helsinki Declaration were followed. The project protocol was approved by the Scientific and Ethics Committee of our institution. All women participating in this study were given medical attention at the highest level of medical specialties in a health center in Mexico City (with ethical approval code number 212250-49501).

## 5. Results

Of the 95 mothers with a high-risk pregnancy and 32 with a normal pregnancy, the median age was 28 years (IQR = 20–34 years), cohabitation was the most common marital status, and homemaker was the most common occupation. Regarding educational level, nearly 90% had finished middle school. Concerning obstetric background, 44% of the participants were primigravida, 98% of the births took place by caesarean section, and the average weight of the neonates was 1686 g (Table 1).

**Figure 1:**
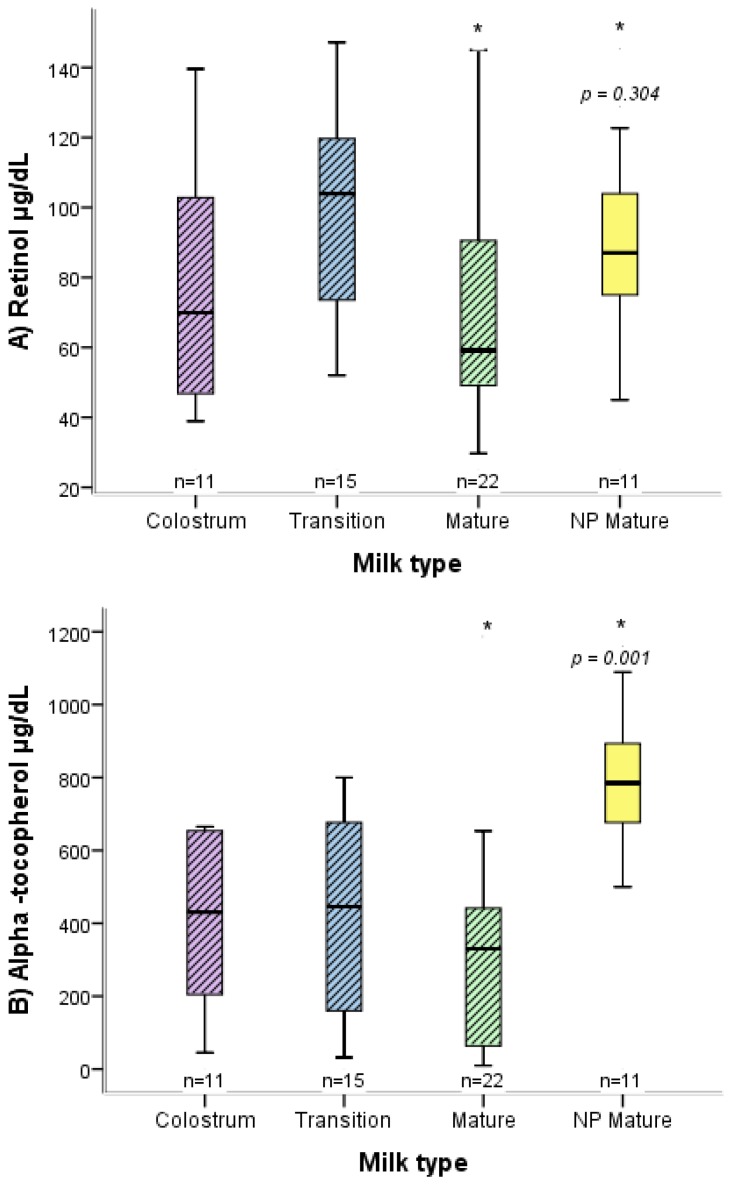
Box plot distribution of the concentration of retinol (**A**) and α-tocopherol (**B**) in breast milk, comparing the women with a full-term, high-risk pregnancy to those with a normal pregnancy. For the high-risk pregnancy, the distinct types of breast milk are shown (colostrum, transition milk, and mature milk). * *p*; Mann-Whitney *U*. The bottom and top of the box represent the first and third quartiles (IQR), and the band inside the box the median. The ends of the whiskers denote the lowest and highest values still within 1.5 IQR. Outlier values are not displayed. NP, normal pregnancy.

**Figure 2:**
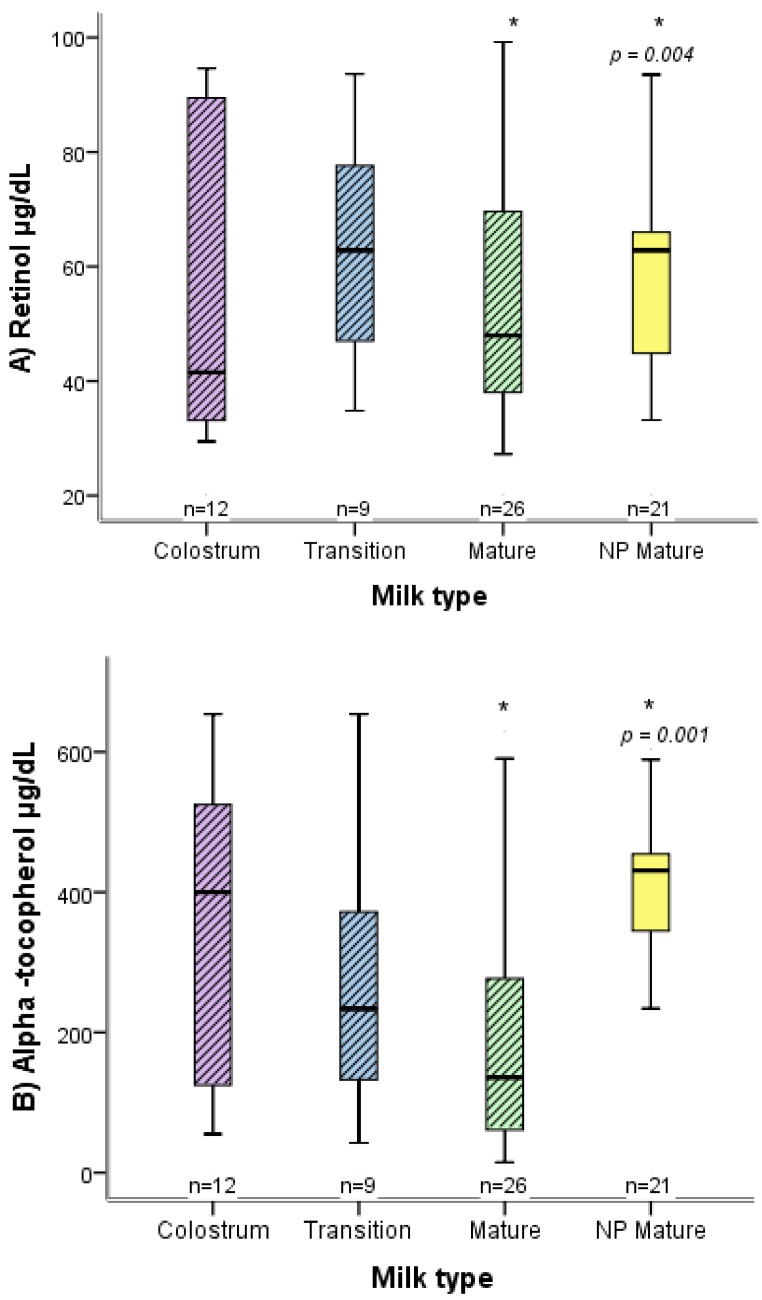
Box plot distribution of the concentration of retinol (**A**) and α-tocopherol (**B**) in breast milk, comparing women with a preterm, high-risk pregnancy to those with a normal pregnancy. For the high-risk pregnancy, the distinct types of breast milk (colostrum, transition milk and mature milk) are shown. * *p*; Mann-Whitney *U*. The bottom and top of the box represent the first and third quartiles (IQR), and the band inside the box the median. The ends of the whiskers denote the lowest and highest values still within 1.5 IQR. Outlier values are not displayed. NP, normal pregnancy.

There were differences between the two groups of mothers (normal versus high-risk pregnancy) in relation to the concentration of retinol and α-tocopherol in mature breast milk (Figure 1 and Figure 2), as well as in neonate weight and length. Concerning anthropometric characteristics, women with a high-risk pregnancy had an average pregestational BMI of 25.5 kg/m^2^ and those with a normal pregnancy 24.5 kg/km^2^, while the average gestational weight gain was 8.2 kg for both groups (Table 1).

For the women with a high-risk pregnancy, the most common disorder or condition related to risk was preeclampsia (40/95), followed by anemia (29/95), advanced maternal age (14/95), and gestational diabetes (12/95). The complications of the neonates born of these women were preterm birth or low birth weight (62/95), infant respiratory distress syndrome or gastroschisis (18/95), and lesions related to the birth canal (6/95). The rest showed no complications (9/95).

When comparing the distinct subgroups of women with different disorders during gestation, there were similar concentrations of retinol and α-tocopherol in breast milk (Table 2). However, the concentration of retinol and α-tocopherol in breast milk was lower in women that gave preterm birth compared to those who carried their pregnancy to term. Considering the women with a high-risk pregnancy, the concentration of retinol was higher in the mature milk of women with normal pregestational weight, while no significant difference was found in the concentration of α-tocopherol among those having adequate gestational weight gain.

Women with normal pregestational BMI had a higher concentration of retinol compared to those who were overweight or obese (see Table 2). The Spearman correlation revealed that the greater the pregestational weight (relative to normal), the lower the concentration of retinol (*Rho* = −0.280, *p* = 0.006). Moreover, there was a correlation between α-tocopherol and retinol (*Rho* = 0.463, *p* = 0.001).

Compared to women with a normal pregnancy, the mature breast milk of those with a high-risk pregnancy who delivered at full term showed a similar concentration of retinol but a lower concentration of α-tocopherol. Compared to the mature milk of women with a normal pregnancy, in contrast, the breast milk of those with a high-risk pregnancy who delivered preterm had a reduced concentration of retinol and α-tocopherol in the diverse types of milk (colostrum, transition milk, and mature milk) (Figure 1 and Figure 2).

When the logistic regression model included variables that corresponded to a high-risk pregnancy, it was observed that in all cases the concentration of both vitamin precursors was lower with high-risk versus normal pregnancy. However, no particular risk factors were associated with these lower concentrations. Hence, the following model was performed.

For the regression model, we utilized the median values for concentrations of retinol below 60 µg/dL and for α-tocopherol below 276 µg/dL in the group high-risk pregnancy, while using these values below 76 and 673 µg/dL for retinol and α-tocopherol, respectively, in the group with a history of normal pregnancy. For the high-risk pregnancy group, the variables associated with a low concentration of retinol in breast milk were preterm childbirth and pregestational overweight or obesity. For the normal pregnancy group, only pregestational overweight or obesity was significant. Meanwhile, for all the women (whether having a high-risk pregnancy or a history of normal pregnancy), a significant risk of a low concentration of α-tocopherol was correlated with preterm childbirth and a tendency was associated with preeclampsia (Table 3).

Regarding the different types of breast milk (Table 4), the concentration of both α-tocopherol and retinol were lower among women who gave preterm childbirth. A positive correlation existed in all cases between the concentration of retinol and that of α-tocopherol in the different types of milk, with *Rho* values of 0.325 for colostrum, 0.375 for transition milk, and 0.300 for mature milk (*p* ≤ 0.001).

There was a reduced level of retinol and α-tocopherol in mothers giving preterm birth or having low birth-weight neonates compared to those that carried their pregnancy to term. However, the concentration of retinol (*p* = 0.244) and α-tocopherol (*p* = 0.090) did not show a significant difference in relation to complications of the newborn. Likewise, no significant difference existed for the concentration of retinol (*p* = 0.243) or α-tocopherol (*p* = 0.897) in regard to the history of pregnancies.

When comparing women with preterm delivery to those who carried their pregnancy to term, the concentration of retinol was 54 (IQR; 37–78) vs. 70 (IQR; 49–106) μg/dL (*p* = 0.002), while that of α-tocopherol was 155 (IQR; 74–410) vs. 445 (IQR; 160–500) μg/dL (*p* = 0.053). On the other hand, compared to women with a high-risk pregnancy, those with a normal pregnancy had a higher level of both vitamin precursors in mature milk.

## 6. Discussion

There was an association between a lower concentration of retinol and α-tocopherol in mature breast milk and two risk factors: pregestational overweight or obesity and preterm delivery. This significance was established with the original statistical analysis and confirmed by the logistic regression model. No significant difference in the concentration of retinol or α-tocopherol was found in relation to either of the morbidities existing among women in the high-risk pregnancy group. Although no direct association was detected between preeclampsia and lower levels of these vitamin precursors, this morbidity is related to overweight and obesity, a condition that, in turn, may cause preterm delivery.

### 6.1. Concentration of Retinol and α-Tocopherol

Considering that breast milk is the only source of nutrition for newborns, it is important to determine whether the content of vitamins A and E are adequate, especially when the neonate is premature or of very low weight [25,26]. In the majority of reports, the concentration of α-tocopherol in the colostrum of women is two to three times higher than that detected presently in Mexican women after a high-risk pregnancy. For the women with a normal evolutive pregnancy, some of the current results are similar to those described for women from Bangladesh and Poland [27,28].

Concerning retinol, for the group of mothers in this study with a history of normal pregnancy and having a normal delivery, the average concentration of this vitamin precursor is close to that commonly reported. In contrast, the women in the present study with a high-risk pregnancy had, on the average, about half the concentration of retinol previously found for women with a normal pregnancy, and a third of this concentration in the cases of preterm childbirth. These values are close to those observed in a group of women in Bangladesh [27,29,30].

The similarities in the concentration of these two vitamins between Mexico and Bangladesh [27] may be due to the characteristics of the particular populations under study. That is, the women in both studies had a low socioeconomic status and poor health indicators, which makes them more prone to the development of nutritional deficiencies. Although the nutrient concentrations found in the women are low, Ahmed et al. demonstrated that this amount is sufficient to satisfy the nutritional needs of infants.

In relation to transitional milk, the concentration of retinol and α-tocopherol described in diverse populations [31,32,33,34,35] is higher than that evidenced in the present study after a high-risk pregnancy. Kodentsova and Vrzhesinskaya reported a mean concentration for each of these vitamin precursors in Russian women that was very similar to the present high-risk pregnancy group. This similarity is likely due to the fact that approximately 50% of Russian women who participated in the study experienced a preterm pregnancy [36].

Regarding mature milk in the current contribution, the mean concentration of α-tocopherol and retinol for both groups of women (with a normal pregnancy and high-risk pregnancy) is very similar to most reports on these micronutrients. However, for women who, herein, had a high-risk pregnancy, the median concentration of α-tocopherol was higher than that detected in Polish women [37,38,39,40,41,42,43] and in a group of adolescent mothers in Brazil [44]. In another study by Tokusoglu et al. [45], the concentration of α-tocopherol in the mature milk of Turkish women was similar to that commonly observed in colostrum. Quiles et al. [26] found higher than average values for mature milk in Spain, but this was based on only 15 women. The small sample size likely influenced the results.

The level of α-tocopherol and retinol in breast milk is normally greater in colostrum and thereafter exhibits a downward trend during the different stages of lactation. In this study, the women with a high-risk pregnancy showed this downward trend. We compared the average concentration of α-tocopherol previously reported for different groups of women, mostly with a normal evolutive pregnancy, to that found presently for women with a high-risk pregnancy. This concentration was herein found to be 2–3 times lower in colostrum, approximately two times lower in transitional milk, and very similar in mature milk [31,32,46,47,48].

Although an inverse correlation has been reported between the concentration of retinol and α-tocopherol in colostrum [49], the current results evidence a positive correlation between these two vitamins, which implies the existence of suboptimal concentrations. This manifests the need for supplementation [50] to prevent deficiencies in any given population.

### 6.2. Preterm Childbirth

For women in the present study with preterm birth, the median concentration of 405 (IQR; 109–676) µg/dL of α-tocopherol in colostrum was lower than that detected in the colostrum of some other groups of women having the same gestational age. For example, there were 1450 µg/dL in a group of German women [51], 1292.1–1722.8 µg/dL in Spanish women [26], and 1222 ± 772 µg/dL in Tunisian women [14]. Nevertheless, the current findings coincide with the concentration of α-tocopherol in the colostrum of women in other studies that gave preterm birth, such as the 250 µg/dL detected in Canadian women [52] and the 260.0 ± 30.0 µg/dL for Russian women [31]. In the case of retinol in the colostrum of women having given preterm birth, the median value of 36 (32–63) µg/dL observed herein is lower than the 57.5 ± 50.1 µg/dL described by Fares et al. [14]. This suggests the need to focus on reinforcing vitamin levels in women with preterm births and their neonates.

The hypothesis that the concentration of vitamin E depends on gestational age is still very controversial [49,53]. Nonetheless, this idea is supported by the current findings. Similar results were reported for a group of Spanish women by Quiles et al. [26], who found significantly higher concentrations of vitamin E at all stages of lactation in women who had carried their pregnancy to term compared to those giving preterm birth.

Compared to the women that carried their pregnancy to term in the present study, those that gave preterm birth had lower concentrations of retinol and α-tocopherol. The greatest deficiency was detected for the latter vitamin precursor. These results coincide with the findings of Souza et al. [54]. To compensate for this deficiency, supplementation of these vitamins should be provided during pregnancy (especially when involving high-risk) and breastfeeding. Unlike the current results, a Brazilian study reported variable concentrations of α-tocopherol, without any difference between women giving premature birth and those carrying their pregnancy to term. It has been postulated that the concentration of this vitamin is associated with birth weight more than prematurity [53]. However, this idea was not corroborated presently, evidencing multifactorial causes for the deficiency of this vitamin.

The current findings emphasize the fact that the neonate of a preterm birth requires special care, such as optimal nutrition. Not only does the prematurity of birth imply low reserves of vitamins A and E, but also the poor nutritional status of the mother indicates a limited transfer of the same through the placenta. For these reasons, liposoluble vitamins (e.g., retinol and α-tocopherol) should be assimilated by women with high-risk pregnancies [55] to avoid complications such as a delay in intrauterine growth or in neurodevelopment. A deficiency of these vitamins can even induce miscarriage [29].

Furthermore, it is recommended that preterm neonates immediately begin enteral feeding with breast milk and, if possible, vitamin supplementation [55]. Indeed, clinical assays have been conducted in which pregnant women were given a supplement of RRR α-tocopherol acetate, resulting in a significantly elevated concentration of this vitamin in transition milk and mature milk, as well as in the mothers themselves who gave preterm birth [50].

### 6.3. Does the BMI Affect the Concentrations of Retinol and α-Tocopherol?

The current results reveal that the higher the pregestational BMI (relative to normal) and the greater the gap between the real and recommended gestational weight gain, the lower the concentration of retinol tends to be. In this sense, the simple fact of having a high BMI is a risk factor for increased production of proinflammatory cytokines, a problem that can be counteracted if women are protected by adequate concentrations of two antioxidant substances, retinol and α-tocopherol. Perhaps it was the deficiency of retinol and α-tocopherol that, in large part, caused the relatively high frequency of preeclampsia among the women presently studied, considering that this deficiency (as well as others) can lead to greater oxidative stress [37] which, in turn, when coupled with other factors, tends to trigger this disorder [56,57].

A negative correlation was observed herein between pregestational BMI and the concentration of retinol and α-tocopherol. Like any population with overweight and obesity, pregnant women lack antioxidants and are exposed to cytokines that can harm their health and that of their offspring, thus affecting fetal programming and the first 1000 days of life [58,59,60].

The results suggest that the present participants showed no association between gestational diabetes (or other morbidities correlated with pregnancy) and low concentrations of retinol and α-tocopherol in breast milk, in agreement with another report that found no such association [11]. Since breast milk is the only source of these vitamins, however, there may be a greater risk, especially in the case that the newborn is premature or suffers from complications that increase oxidative stress.

The current results, like the vast majority of studies on liposoluble vitamins in breast milk [61,62,63], evidence a wide range in the concentration of vitamins A and E (especially vitamin E) among participants. This variation could be related to differences in lifestyle, in the consumption of nutrients and/or enrichment of food, and in the use of vitamin and food supplements. Some methodological factors, such as the day and time of collecting samples or the time elapsed since the last breastfeeding and the last meal, could contribute to this variability. Additionally, differences in methods of analysis also represent an important factor of variation. Finally, the methods employed for hydrolysis and extraction of an organic matrix of vitamins can affect the concentration of the same in breast milk [62].

Nourishing an infant with milk from his or her own mother results in better growth and nutritional status (including the level of vitamins A and E) than that observed in infants fed formula. Moreover, breast milk composition may be affected by obstetric characteristics, such as a premature delivery. In the present study, the composition of retinol and α-tocopherol in breast milk was negatively affected by premature birth and pregestational overweight and obesity. Furthermore, the concentration of these vitamin precursors was lower in the breast milk of women that had undergone a high-risk pregnancy compared to those having had a normal pregnancy.

Studies that explore the association between neonatal/maternal characteristics and the concentration of retinol and α-tocopherol in breast milk are important for determining the subgroups of newborns at risk for a vitamin A and E deficiency. This information should lead to strategies (e.g., vitamin supplementation) to target the subgroups at risk. Moreover, this approach can broaden the understanding of the influence of maternal factors such as gestational age on the adaptation capacity of mechanisms for transferring retinol and α-tocopherol to the mammary gland.

Certain elements of this study could be considered as limitations, including the lack of an evaluation of the nutritional state and diet of the participants. Another limitation is that a determination of fat in breast milk samples was not made. However, in other populations a high negative correlation has been shown between the concentration of serum cholesterol, the BMI, and the level of tocopherol and retinol. In addition, it must be kept in mind that all of the women participating in this study were of a low socioeconomic level, meaning that the results obtained cannot be generalized to all Mexican women during the period of breastfeeding.

## 7. Conclusions

Since the nutritional needs of a woman are sharply increased during pregnancy, it can be a challenge to meet these needs if careful attention is not paid to an adequate consumption of nutrients. Any inadequate dietary alteration can affect the health of the mother-fetus binomial. Deficiencies in micronutrients are related to gestational diabetes, preeclampsia, eclampsia, delayed intrauterine growth, low birth weight of the neonate, premature separation of the placenta, premature childbirth, spontaneous abortion, and congenital anomalies. Moreover, maternal nutrition determines the quality of breast milk. The concentration of vitamins A and E in breast milk depend on the concentration of the same in the mother. Hence, a maternal deficiency translates into the same problem for the neonate.

Even though the women in the high-risk pregnancy group showed a deficiency of vitamins A and E in their breast milk, the unique biological benefits of this milk justify the promotion of breast feeding as the optimal method of nourishing neonates. In these cases, it should be recommended that the woman increase her consumption of certain nutrients during pregnancy. Additionally, after childbirth, she should consider the use of supplements to produce milk of adequate quality and, thus, meet the needs of the baby and prevent a deficiency of micronutrients.

When the concentration of vitamins A and E are low in the breast milk of mothers after a high-risk pregnancy, we also recommend supplements for a child who receives nutrition exclusively from the mother. This supplementation should begin during the first days of life and continue until the infant initiates a complementary diet. Some studies have demonstrated that a deficiency in vitamins A and E in newborns, above all those with premature birth, increases the frequency of cases of bronchopulmonary dysplasia, intraventricular hemorrhage, periventricular leukomalacia, retinopathy, and necrotizing enterocolitis. Currently, there is not enough information to evaluate the possible benefits or adverse effects of supplementation during pregnancy.

In the current study, maternal overweight and obesity, as well as preterm birth, were associated with a low concentration of vitamins A and E in breast milk. It is necessary to determine whether the level of physical activity and the individual metabolism influence the quantity of food that each woman needs to achieve an optimal nutritional state and an adequate production of milk. The length and intensity of lactation also significantly affects the nutritional needs of the mother, although this is rarely considered.

Finally, during the period of breast feeding both the mother and child are at risk for a deficiency of vitamins A and E, especially in developing countries like Mexico and, thus, represent a public health problem. Moreover, if the concentration of vitamins A and E are low in the mother, the child is susceptible to suffering a like deficiency.

This study improves the knowledge about the content of vitamins A and E in breast milk from mothers after a high-risk pregnancy. Future research could include other nutrients that are also important for the development of the breast-fed child, as well as evaluate the nutritional intake of women during a high-risk pregnancy.

## Figures and Tables

**Table 1:** Clinical, anthropometric and sociodemographic characteristics of the mothers and newborns (*n* = 127).

Clinical and Anthropometric Characteristics			
	High-risk pregnancy *n* = 95	Normal pregnancy *n* = 32	
	Mean ± SD		*p*
Age (years) ^a^	28 ± 5	27 ± 8	0.456
Pregestational weight (kg) ^a^	62.2 ± 10	59.9 ± 10	0.253
Maximum gestational weight (kg) ^a^	70.4 ± 13	67 ± 13	0.239
Height (cm) ^a^	156 ± 6	156 ± 5	0.632
Pregestational BMI ^a^	25.5 ± 4	24.5 ± 3	0.176
Number of prenatal check-ups ^a^	4 ± 2	4 ± 0.5	0.211
Gynecological age (years) ^a^	15 ± 8	16.5 ± 5	0.890
Menarche (age in years) ^a^	12 ± 2	12 ± 1	0.878
Weight of the newborn (g) ^a^	1686 ± 843	2167 ± 689	0.004
Length of the newborn (cm) ^a^	40 ± 5	48.2 ± 5	0.006
Sociodemographic characteristics ^b^	Frequency (%)		
Marital status			
Single	23 (24)	4 (13)	0.556
Married	35 (37)	11 (34)	
Cohabitation	37 (39)	17 (53)	
Occupation			0.883
Homemaker	73 (77)	24 (75)	
Working outside the home	22 (23)	8 (25)	
Level of education			0.823
Primary or less	10 (11)	2 (6)	
Middle school	40 (42)	14 (44)	
High school	33 (35)	13 (41)	
Professional	12 (12)	3 (9)	
Socioeconomic level			0.983
Lower to lower-middle class	95 (100)	31 (96)	

Data expressed as the ^a^ mean ± standard deviation—Student’s *t*; ^b^ frequency (%)—Pearson’s *X*^2^.

**Table 2:** Concentrations of retinol and α-tocopherol in breast milk, in relation to some perinatal data of the women with a high-risk pregnancy (*n* = 95).

Retinol						α-Tocopherol		
		*n*	Colostrum µg/dL	Transition Milk µg/dL	Mature Milk µg/dL	Colostrum µg/dL	Transition Milk µg/dL	Mature Milk µg/dL
			Median (IQR)	Median (IQR)	Median (IQR)	Median (IQR)	Median (IQR)	Median (IQR)
Maternal age (years)	≤19	21	73 (37–93)	57 (52–70)	67 (49–70)	500 (103–654)	276 (55–445)	207 (48–270)
	20–29	29	67 (35–128)	84 (64–103)	60 (49–82)	298 (127–492)	204 (103–321)	321 (68–436)
	30–34	23	42 (33–50)	93 (64–120)	42 (35–56)	500 (405–597)	654 (348–727)	103 (46–255)
	≥35	22	79 (52–106)	62 (39–81)	67 (49–70)	275 (192–570)	201 (167–475)	339 (180–423)
*p* ^a^			0.225	0.340	0.173	0.632	0.407	0.330
Pregestational BMI	Normal weight	41	60 (47–96)	80 (66–97)	60 (42–80)	575 (154–654)	180 (131–410)	321 (55–448)
	Overweight	40	60 (38–94)	64 (41–93)	45 (34–64)	321 (130–540)	249 (90–450)	190 (54–340)
	Obesity	14	33 (33–33)	42 (32–52)	49 (38–55)	455 (410–500)	295 (146–445)	146 (103–423)
*p* ^a^			0.209	0.095	0.003	0.575	0.945	0.620
Gestational weight increase	Adequate	19	31 (29–33)	72(44–120)	50 (34–60)	270 (130–410)	549 (323–727)	340 (61–423)
	Low	35	60 (42–93)	73 (70–110)	62 (49–90)	470 (120–654)	180 (107–433)	218 (63–339)
	Excessive	41	61 (45–94)	64 (53–89)	55 (40–79)	400 (253–602)	249 (103–372)	180 (103–423)
*p* ^a^			0.090	0.136	0.337	0.802	0.911	0.951
Gestational age	To term	47	60 (46–102)	103 (73–120)	59 (49–91)	470 (275–654)	445 (160–676)	330 (63–441)
	Preterm	48	41 (33–89)	62 (47–77)	48 (38–70)	400 (125–525)	132 (233–371)	135 (61–276)
*p* ^b^			0.069	0.005	0.058	0.260	0.290	0.118
Complication during pregnancy	Gestational diabetes	12	49 (44–93)	94 (70–119)	47 (35–99)	400 (321–500)	433 (189–676)	38 (18–431)
	Preeclampsia	40	60 (41–92)	62 (40–76)	59 (46–76)	343 (230–654)	233 (115–445)	218 (104–450)
	Anemia, myomas	29	49 (32–83)	93 (70–103)	47 (35–67)	545 (125–654)	321 (160–450)	205 (59–416)
	Advanced maternal age	14	90 (62–96)	76 (52–100)	68 (49–85)	360 (183–500)	274 (103–445)	180 (103–321)
*p* ^a^			0.690	0.260	0.378	0.905	0.906	0.661

^a^ Kruskal-Wallis; ^b^ Mann-Whitney *U*; IQR, interquartile ranges.

**Table 3:** Variables associated with low concentrations of retinol and α-tocopherol in mothers that underwent a high-risk pregnancy (*n* = 95).

Maternal variables	The Median Concentration in Mature Milk of Mothers Who Underwent a High-Risk Pregnancy			The Median Concentration in Mature Milk of Mothers Who Experienced a Normal Pregnancy		
	OR	CI 95%	*p* *	OR	CI 95%	*p* *
	Retinol concentration (≤60 µg/dL)				(≤76 µg/dL)	
Preterm childbirth	2.618	1.111–6.169	0.028	2.112	0.858–5.856	0.112
Preeclampsia	1.512	0.429–5.450	0.512	0.992	0.992–1.138	0.102
Gestational diabetes	0.879	0.277–3.122	0.575	0.272	0.023–3.249	0.273
Anemia	0.877	0.281–3.629	0.258	0.955	0.906–1.006	0.331
Maternal age: under 19 or over 29 years	0.818	0.364–1.838	0.390	2.189	0.858–5.586	0.101
Pregestational overweight or obesity	1.179	1.822–2692	0.039	3.563	1.422–8.927	0.007
	α-tocopherol concentration (≤276 µg/dL) ^a^			(≤673 µg/dL) ^b^		
Preterm childbirth	2.243	0.985–5.111	0.039	2.091	1.689–2.588	0.038
Preeclampsia	1.304	0.567–3.001	0.532	1.063	0.992–1.138	0.051
Gestational diabetes	0.892	0.228–3.483	0.869	0.289	0.023–2.951	0.336
Anemia	0.914	0.252–3.319	0.892	0.955	0.906–1.006	0.331
Maternal age: under 19 or over 29 years	1.879	0.459–2.484	0.879	0.402	0.035–4.591	0.441
Pregestational overweight or obesity	1.056	0.447–2.494	0.870	0.772	0.064–9.302	0.838

CI 95%: 95% confidence interval. OR: odds ratio. Low concentrations of retinol and α-tocopherol: value below the median in mothers after a high-risk pregnancy and those with a history of a normal pregnancy. * *p* Value in logistic regression model.

**Table 4:** Concentration of retinol and α-tocopherol in relation to the type of breast milk.

**Pregnancy Length**	**Colostrum**	**Transition**	**Mature**
	**Retinol, median (IQR), µg/dL**		
Preterm (*n* = 48)	36 (32–63)	60 (39–81)	43 (36–66)
Full term (*n* = 47)	49 (39–86)	73 (49–115)	52 (37–72)
*p* ^a^	0.010	0.020	0.032
	**α-tocopherol, median (IQR), µg/dL**		
Preterm (*n* = 48)	405 (109–676)	233 (90–372)	175 (59–465)
Full term (*n* = 47)	654 (205–2313)	676 (174–1884)	285 (55–477)
*p* ^a^	0.004	0.001	0.012

Data expressed as the median (IQR). ^a^ *Mann Whitney U*; IQR, interquartile range.

## Abbreviations

HPLC	High-performance liquid chromatography
BMI	Body mass index
WHO	World Health Organization
PAHO	Pan American Health Organization
IQR	Interquartile range
